# Italy’s Post-COVID-19 Stroke Network: Has It Returned to Pre-Pandemic Standards?

**DOI:** 10.3390/epidemiologia5030025

**Published:** 2024-07-09

**Authors:** Erika Kacerik, Francesca Bottega, Aida Andreassi, Giuseppe Sechi, Alberto Zoli, Marco Botteri, Carlo Signorelli, Nazzareno Fagoni

**Affiliations:** 1 Faculty of Medicine, School of Public Health, University of Vita-Salute San Raffaele, Via Olgettina 60, 20132 Milano, Italy; 2 Agenzia Regionale Emergenza Urgenza Headquarters (AREU HQ), Via Campanini 6, 20124 Milano, Italy; 3 Department of Molecular and Translational Medicine, University of Brescia, 25123 Brescia, Italy

**Keywords:** emergency department, COVID-19, stroke, Lombardy region

## Abstract

The COVID-19 pandemic strongly transformed the healthcare system in the Lombardy region (Italy), forcing a rapid reorganization of hospital structures. The emergency medical service (EMS) system and emergency departments (EDs) were among the most affected departments. Several studies have shown a change in the epidemiology of time-dependent pathologies, such as stroke, during the pandemic’s peak. However, there is little scientific evidence regarding the interpandemic phase. The regional register for ED accesses (EUOL) was analyzed, taking into consideration all accesses for stroke and stroke-like syndromes during the years 2019, 2020, and 2021. The analysis shows a significant difference in the average number of diagnoses per month [2092 vs. 1815 vs. 2015, respectively (*p* < 0.05)] and an increase in the percentage of transports carried out by EMS vehicles to EDs [17% vs. 25% vs. 22%, respectively (*p* < 0.05)]. The length of stay (LOS) increased for both discharged patients (9.0 vs. 10.1 vs. 11.2 h, respectively; *p* < 0.005) and hospitalized patients (7.5 vs. 9.7 vs. 10.6 h, respectively; *p* < 0.005). During the COVID-19 pandemic, the overall number of stroke diagnoses decreased, while the percentage of patients transported to EDs by EMS vehicles increased. Furthermore, an increased processing time in EDs was highlighted.

## 1. Introduction

Stroke is a leading cause of morbidity and mortality worldwide, presenting a significant burden on healthcare systems [1]. Stroke and stroke-like syndromes, although similar in presentation, differ significantly in their underlying pathophysiology and clinical management. Stroke is characterized by an acute interruption of blood flow to the brain leading to ischemic or hemorrhagic events, which require immediate intervention to restore perfusion and minimize neuronal damage [2]. In contrast, stroke-like syndromes often mimic stroke symptoms, but do not result in permanent brain damage [3]. Accurate differentiation between these conditions is crucial for appropriate treatment and secondary prevention strategies.

The stroke network underwent significant modifications during the COVID-19 pandemic, as evidenced by the decrease in the number of diagnoses [4,5] made in emergency departments (EDs) and the increase in patient transport time from home to hospital [6]. The epidemiology of stroke markedly changed [4] for various reasons. Public health communication proved to be a vital tool, conveying messages that were demonstrated to have rapidly altered the population’s attitudes [7,8,9]. As a result, the “stay at home” policy, communicated by the Italian Ministry of Health, prompted many citizens to refrain from accessing EDs [10] and seeking medical care. Another significant aspect is that, during the pandemic, there were also changes in the incidence and distribution of the disease. Patients with COVID-19 have shown a higher incidence of stroke that developed as a complication of the infection [11,12,13].

The prevalence of strokes in patients with COVID-19 has been a significant concern, with studies indicating an elevated risk due to both direct viral damage to brain tissue and the exacerbation of pre-existing comorbidities [11,14,15]. According to the research, SARS-CoV-2 can induce hypercoagulability and inflammation, leading to increased instances of cerebrovascular events [14]. Additionally, comorbid conditions, such as hypertension and diabetes, common in COVID-19 patients, further elevate the risk of stroke, contributing to a higher incidence of severe neurological outcomes [15]. Moreover, some neurological symptoms of COVID-19, such as confusion or difficulties with speech, can be misinterpreted by emergency medical services (EMS) system operators as signs of stroke [11,12,13].

Italy was the first European country to be heavily affected by COVID-19 and to adopt restrictive measures against the pandemic [16,17]. Following the identification of the first case in Italy on 21 February 2020, initial lockdown measures were implemented starting from 23 February 2020, initially targeting only a restricted geographical area. In response to the evolving epidemiological situation, the lockdown was extended to the entire national territory on 9 March 2020 [16,17]. The lockdown significantly impacted all organizational areas, including the healthcare sector and the EMS system [18,19,20], leading to changes in professional training [21], EMS transport modalities [22], the number of ED accesses and hospitalizations [23], and clinical characteristics of patients [22]. The Lombardy region, the most populated Italian region, with approximately 10 million residents, hosts 218 hospitals, 102 of which are equipped with an ED. Every ED in the Lombardy region registers ED accesses on the EUOL (“Emergenza Urgenza OnLine”—Emergency Urgency OnLine) portal [24].

The territorial emergency–urgency system of the Lombardy region is coordinated by the Agenzia Regionale Emergenza Urgenza (AREU—Regional Emergency Urgency Agency), a single institution responsible for coordinating the entire emergency transport system. Throughout various phases of the pandemic, the AREU had to respond swiftly by implementing protocols for the management of COVID-19 patients and increasing the number of rescue vehicles to maintain pre-pandemic first response standards [25,26].

The main purpose of our study was to analyze epidemiological changes in the stroke network during pre- and post-pandemic phases. Our hypothesis was that, with the conclusion of the pandemic phase, all indicators have returned to values equivalent to those of the pre-pandemic period.

## 2. Materials and Methods

This was a retrospective observational cohort study. All information was anonymous and there were no sensitive patient data.

Data for this study were provided by the EUOL portal for the years 2019, 2020, and 2021. We analyzed the number of diagnoses of stroke and stroke-like pathologies, the severity level assigned during triage (using a color code, in which “red” indicates emergency, “yellow” indicates urgency, and “green” and “white” are non-urgent), demographic characteristics of patients (sex and age), mode of arrival to an ED (autonomous, EMS system, helicopter rescue), the outcome at discharge from an ED (e.g., hospitalized or discharged to home), the length of stay in an ED (LOS, defined as the cumulative time elapsed between admission and discharge from an ED), and the diagnosis at discharge, which was established by an ED medical doctor. According to ICD9-CM codes [27], this study encompassed the following stroke diagnoses: subarachnoid hemorrhage (430.X), intracerebral hemorrhage (431.X), other and unspecified intracranial hemorrhage (432.X), and occlusion of cerebral arteries (434.X). For stroke-like pathologies, this study included transient cerebral ischemia (435.X), acute but ill-defined cerebrovascular disease (436.X), and other and ill-defined cerebrovascular disease (437.X). Overall, this study included 71,016 patients with stroke and stroke-like syndromes.

We considered three periods for the definition of the first, second, and third pandemic waves in Italy: March 2020, November and December 2020, and February and March 2021, respectively.

Categorical variables are presented as numbers, and continuous variables are presented as means and standard deviations. Continuous variables were tested for normality by means of the Kolmogorov–Smirnov test, were analyzed by ANOVA testing, and relative odds ratios (OR) and confidence intervals (with 95% CI) were calculated. Differences were considered significant when *p* < 0.05, otherwise they were considered non-significant (NS). Prism 8.0.1 statistical software (GraphPad Software LLC, San Diego, CA, USA) was used for this aim.

## 3. Results

In line with the purpose of this study, we systematically gathered all recorded ED admissions from the EUOL database with diagnoses of stroke and stroke-like syndromes. Notably, this dataset was restricted solely to patients who arrived at an ED, and therefore it does not include all patients treated by EMS. Moreover, it is important to emphasize that the Lombardy region had around 3 million ED admissions each year.

In the three years under examination, differences were recorded regarding admissions to EDs in the Lombardy region for patients diagnosed with stroke or stroke-like syndromes.

According to data presented in Table 1, the total number of individuals accessing EDs with a diagnosis of stroke or stroke-like syndromes amounted to 25,085 subjects in 2019, which decreased to 21,764 in 2020. However, in 2021, the number escalated to 24,167 admissions. Patients generally presented in more severe condition upon arrival at an ED, as evidenced by the increasing percentage of red (emergency) codes during triage over the three reference years (22.2% in 2019 vs. 24.5% in 2020 vs. 25.9% in 2021). Hospitalization remained the most predominant outcome for these patients, with rates of 64.2% in 2019, 64.3% in 2020, and 62.4% in 2021.

**Figure 1:**
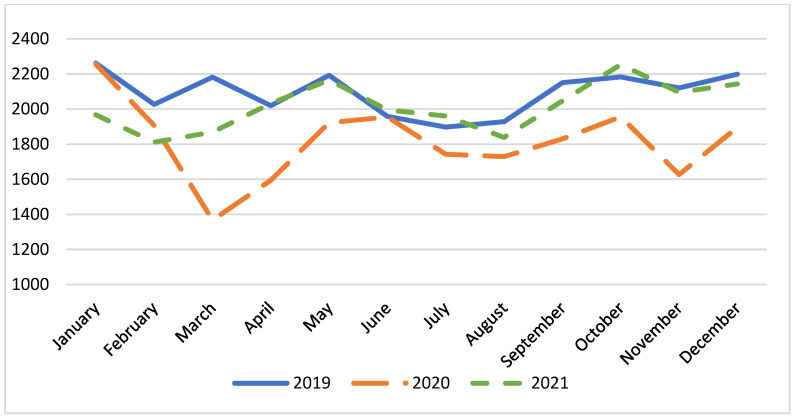
Monthly numbers of patients with stroke or stroke-like syndromes who accessed an ED.

Figure 1 shows the monthly incidence of patients with stroke and stroke-like syndromes who accessed EDs; this value significantly decreased, passing from 2092 in 2019 to 1815 in 2020, and 2015 in 2021 (*p* < 0.001). Notably, the three lowest average number of diagnoses occurred in March 2020 (1367), November 2020 (1627), and February 2021 (1811), corresponding exactly to the three pandemic waves. The minimum number of diagnoses was recorded during the first wave; however, given the total number of ED accesses, the likelihood of presenting in March 2020 with stroke or stroke-like syndromes was higher, with an OR of 1.50 [95% CI, 1.38 to 1.58, *p* < 0.0001].

**Figure 2:**
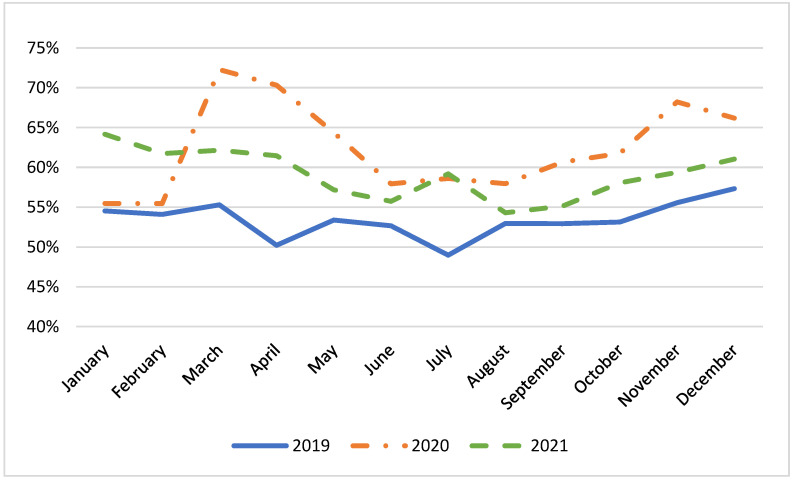
Percentages of patients with stroke or stroke-like syndromes transported by EMS.

Figure 2 depicts the number of patients with stroke and stroke-like syndromes who arrived at EDs via the EMS system. The monthly average increased from 53% in 2019 to 63% in 2020 and 59% in 2021 (*p* < 0.0001). The percentage peaked at 72% during the first wave. Additional increases were observed during the second (68%) and third (64%) waves.

The length of stay (LOS) is the cumulative time a patient spends in an ED. Table 2 presents the LOS for patients who accessed an ED during the three reference years, 2019, 2020, and 2021. An increased LOS was observed for both discharged patients (9.0 vs. 10.1 vs. 11.2 h, respectively; *p* value < 0.005) and for hospitalized patients (7.5 vs. 9.7 vs. 10.6 h, respectively; *p* value < 0.005). Overall, data indicate an increase in the processing time of patients in EDs for the years 2020 and 2021 compared to 2019.

## 4. Discussion

In line with the purpose of our study, we analyzed the impact of COVID-19 on patients with stroke and stroke-like syndromes accessing EDs in the Lombardy region for the years 2019, 2020, and 2021. We considered in the same population patients with stroke and stroke-like syndromes. The reason for this choice was that patients were not able to distinguish between one pathology and the other at the onset of symptoms; therefore, the call for rescue and modalities of transport should remain the same for both stroke and stroke-like syndromes.

The analysis presented in Figure 1 illustrates a significant reduction in the monthly incidence of diagnoses of stroke and stroke-like syndromes in EDs from 2019 to 2021. The average number of cases decreased by 277 cases per month in 2020 compared to 2019, and in 2021 returned to levels comparable to those in 2019, with a difference of only 77 cases. The decline in the number of diagnoses was particularly evident during the first pandemic wave, in line with the work of Goldberg, S.A. et al. [28]. However, reductions were also observed during the second and third waves, even if there is no evidence in the literature regarding such observations. This trend suggests an impact of the pandemic on healthcare-seeking behavior and hospital accessibility. Stroke is a pathology with a miscellaneous clinical presentation, with symptoms ranging from complete loss of consciousness to subtle neurological signs. We believe that patients with milder symptoms may have avoided showing up at an ED during the pandemic’s peak because they feared being infected by the SARS-CoV-2 virus. This observation is supported by data presented in Table 1, which show that patients with stroke and stroke-like syndromes who presented at an ED had a higher clinical complexity, as indicated by the higher percentage of red (emergency) codes during triage. These findings underscore the dual impact of the pandemic: a reduction in ED visits, possibly due to lockdown measures and fear of virus exposure, and a concurrent increase in the severity of conditions among those who did seek care. Further investigation is required to understand the underlying reasons for these trends and to develop strategies to ensure timely medical care during public health crises.

We observed a significant increase in the percentage of patients with stroke or stroke-like syndromes accessing an ED via EMS vehicles. This percentage reached its maximum value during March 2020 (72%), corresponding with the first pandemic wave. It is important to note that this increase was associated with an overall reduction in the number of accesses to EDs, aligning with data reported by Desai, S.M. [29] and Flamm, A. et al. [5]. The Ministry of Health and other governmental sources repeatedly reiterated the importance of avoiding overcrowding EDs during the pandemic. As a result, there was a pronounced decline in ED visits for less severe conditions. For this reason, the likelihood of diagnosing stroke or stroke-like syndromes for patients who accessed an ED increased when compared to the pre-pandemic period.

The rise in EMS vehicles usage is a noteworthy finding not emphasized by other researchers, to our knowledge. Possible explanations include individuals opting not to have a family member accompany them to an ED due to concerns about COVID-19 transmission, or multiple family members needing simultaneous transport to an ED. This suggests the necessity for increased patient transport resurgence during the early stages of a pandemic, as also highlighted by Gianquinteri et al. [26]. Therefore, national pandemic plans should incorporate strategies for augmenting or reallocating resources to ensure the EMS system can swiftly adapt to any potential change in patient demands.

Our analysis indicates that the pandemic had a significant impact on the EMS system, corroborating findings from other studies [30]. Despite EMS personnel being well trained in managing advanced emergencies, such as advanced cardiovascular life support (ACLS) [31] and advanced trauma care [32], protocols for pandemic preparedness and management remain inadequate [33,34,35]. This issue is critical, as EMS personnel must be prepared to confront pandemic scenarios given the substantially different patient profiles encountered during such events compared to the pre-pandemic period.

The length of stay (LOS) represents a patient’s processing time within a particular setting, in this case in an ED. As highlighted in Table 2, an increased LOS was observed for the years 2020 and 2021 when compared to the pre-pandemic period. These data indicate that during the COVID-19 pandemic the emergency–urgency system experienced increased difficulties in processing patients in EDs, as the LOS increased for both discharged and hospitalized patients. This extended LOS can be attributed to several factors associated with the COVID-19 pandemic. Firstly, the increased complexity of triaging and managing patients with potential COVID-19 symptoms likely contributed to longer processing times. Additionally, the strain on healthcare resources, including reduced staffing levels and the reallocation of medical personnel, may have further exacerbated delays. This has critical implications for patient care, as prolonged ED stays can lead to overcrowding, increased risk of adverse events, and decreased patient satisfaction. Furthermore, our data reveal that the LOS in EDs further extended during 2021, a year that, as previously stated, we considered as post-pandemic.

This evidence presented in Table 2 is also crucial because the extension of processing times in EDs during the post-pandemic phase has already been shown in the literature, particularly in studies concerning all ED admissions [36,37,38,39,40,41]; however, our study is the first to demonstrate that this phenomenon also specifically impacted time-dependent pathologies, such as strokes. We believe that specific analyses are necessary to better understand the potential clinical impact of increased processing time in EDs in terms of disability or other outcomes. Moreover, further research should focus on identifying bottlenecks and developing targeted interventions to mitigate the impact of such disruptions on ED operations.

### Strengths and Limitations

A key strength of our study was the extensive time frame analyzed, which included one year of normal activity prior to the pandemic (2019) and two years covering three distinct pandemic waves, with the final wave occurring in early 2021. Consequently, a significant portion of 2021 served as an additional post-pandemic period. Another strength was the large sample size, facilitated by access to the regional database, which includes records from 102 EDs in the Lombardy region and encompassed over 71,000 patients with stroke and stroke-like syndromes over the three reference years.

There were also some limitations to our study as well. The first one is related to the use of administrative and anonymous data for the evaluation of cases. There may have been some missed data or mistakes in recording, especially during pandemic phases, when health workers were deeply involved in the management of patients and working under more pressure than in normal circumstances. Moreover, administrative data flows, established for organizational and monitoring purposes, and entirely anonymized and disconnected from individual patients, have significant limitations in terms of clinical accuracy. Several studies have demonstrated potential inconsistencies between administrative ICD9-CM flows and clinical diagnoses in hospital departments [42,43]. The second aspect is related to the reorganization of the emergency–urgency system: during various pandemic phases, the emergency system endured significant reorganizations, and it is impossible to know how these actions impacted our results.

Post-pandemic epidemiological studies of time-dependent pathologies and their distribution among the population play an important role in determining whether the healthcare system has regained its pre-pandemic level of resilience. Our study aimed to contribute to this disclosure by analyzing a pathology that has a substantial impact on the healthcare system.

## 5. Conclusions

In conclusion, our analysis shows a noteworthy decrease in the overall number of stroke and stroke-like syndromes diagnoses during the pandemic years, alongside an increase in the clinical severity of such patients. Furthermore, the percentage of patients transported to EDs by EMS vehicles significantly increased. Moreover, increases in the LOS show an extension of the processing time in EDs that is still present during the post-pandemic phase, including processing time for time-dependent pathologies.

In conclusion, our results highlight that not all indicators have returned to values comparable to those of the pre-pandemic period. Therefore, these findings underscore the urgent need for improved strategies to manage time-dependent pathologies effectively, even during major public health crises. Ensuring timely medical care and optimizing ED operations are paramount to maintaining resilience in healthcare systems in the face of future pandemics. Future research should focus on identifying specific bottlenecks and developing targeted interventions to improve ED efficiency and patient outcomes. Addressing these challenges is essential to ensure that healthcare systems can better withstand the pressures of public health emergencies and continue to provide high-quality care.

## Figures and Tables

**Table 1:** Demographic characteristics of patients with stroke or stroke-like syndromes.

Characteristics	2019	2020	2021
Diagnoses; N	25,085	21,764	24,167
Gender (Male); N (%)	12,374 (49.3%)	11,170 (51.3%)	12,376 (51.2%)
Age; Average (SD)	74.05 (14.5)	74.09 (14.5)	73.92 (14.9)
Red code during triage; N (%)	5588 (22.2%)	5347 (24.5%)	6257 (25.9%)
Yellow code during triage; N (%)	13,453 (53.6%)	11,501 (52.8%)	12,475 (51.6%)
Hospitalized from an ED; N (%)	16,103 (64.2%)	14,004 (64.3%)	15,080 (62.4%)
Discharged from an ED; N (%)	6350 (25.3%)	5283 (24.3%)	6561 (26.2%)

**Table 2:** The LOS (length of stay) of patients in EDs in hours. * *p*-value < 0.005

	2019	2020	2021
LOS, total access; average (SD)	8.1 (10.6)	9.9 * (12.0)	10.9 * (13.1)
LOS, discharged patients; average (SD)	9.0 (10.2)	10.1 * (11.1)	11.2 * (12.3)
LOS, hospitalized patients; average (SD)	7.5 (10.8)	9.7 * (12.0)	10.6 * (13.1)

## Data Availability

Data presented in this study are available on request from the corresponding author. Data are not publicly available due to national data safety guidelines.
